# High temperature and solar radiation in the Red Sea enhance the dissolution of crude oil from surface films

**DOI:** 10.1007/s11356-024-33864-z

**Published:** 2024-06-10

**Authors:** Sreejith Kottuparambil, Ananya Ashok, Patricia López, Maan H. Amad, Carlos M. Duarte, Susana Agusti

**Affiliations:** 1 Red Sea Research Center (RSRC), King Abdullah University of Science and Technology (KAUST), 23955-6900 Thuwal, Saudi Arabia; 2 Mubadala Arabian Center for Climate and Environmental Sciences (ACCESS), New York University Abu Dhabi, P.O. Box 129188, Abu Dhabi, United Arab Emirates; 3 King Abdullah University of Science and Technology (KAUST), Core Labs, 23955-6900 Thuwal, Saudi Arabia; 4 Computational Bioscience Research Center (CBRC), King Abdullah University of Science and Technology (KAUST), 23955-6900 Thuwal, Saudi Arabia

**Keywords:** Oil spill, Photooxidation, Fluorescence, HNA bacteria, CDOM, PAHs, δ^13^C

## Abstract

The Red Sea is a hotspot of biodiversity susceptible to oil pollution. Besides, it is one of the warmest seas on the Earth with highly transparent waters. In this study, we estimated the oil dissolution rates under natural sunlight spectra and temperature conditions using coastal oil slicks collected after the 2019 Sabiti oil spill in the Red Sea. Optical analyses revealed the significant interactive effect of sunlight and temperature in enhancing the dissolution of oil into dissolved organic matter (DOM). The highest oil dissolution rate (38.68 g C m^−3^ d^−1^) was observed in full-spectrum sunlight. Oil dissolution significantly enhanced total organic carbon (TOC) and polycyclic aromatic hydrocarbons (PAHs) in seawater. High nucleic acid (HNA) bacteria, likely the oil degraders, proliferated from 30 to 70 – 90% after 4 days. The heavier stable carbon isotopic composition of methane (δ^13^C-CH_4_) and lighter stable carbon isotopic composition of carbon dioxide (δ^13^C-CO_2_) indicate the putative role of bacterial processes in the natural degradation of crude oil. The results indicated that the combined effect of temperature and solar radiation enhanced the biological and photochemical dissolution of oil on the Red Sea surface.

**Supplementary Information:**

The online version contains supplementary material available at 10.1007/s11356-024-33864-z.

## Introduction

Oil spills are environmental disasters causing serious marine ecosystem deterioration, public health effects, and socioeconomic impacts on the affected coastal communities (Chang et al. 2014; Huynh et al. 2021; Li et al. 2016). The number of large oil spills and the amount of spilt oil in the world’s oceans have decreased significantly in recent decades (Ritchie et al. 2022). However, smaller spills from ships and operating platforms are frequent and prominent sources of oil contamination along major shipping routes and at oil production sites (Mohr & Gade 2022). Thus, the risk of oil pollution in urban coastal regions has been exacerbated due to the increasing demand for oil, and subsequent oil-related activities and growing population densities (Ferguson et al. 2020).

The fate of marine oil spills is driven by very complex processes, including chemical degradation, biological decomposition, evaporation, and dissolution into the water phase (Passow & Overton 2021). Biodegradation of spilled crude oil is a rapid and extensive process in the marine environment, with an average half-life of 11–14 days, depending on the environmental conditions prevailing in the affected area (Prince et al. 2013). However, exposure to natural sunlight can initiate complex transformation reactions within floating slicks, which accelerate the photodissolution of crude oil by the production of water-soluble polar organic compounds (Freeman & Ward 2022; Harriman et al. 2017). When exposed to sunlight, crude oil hydrocarbons degrade through three concerted processes: photooxidation, photofragmentation, and polymerization (Chacón-Patiño et al. 2020). Depending on the location of the spill, a substantial amount of the spilled oil can also be transported to the shoreline, by currents and waves causing prolonged re-oiling of the beaches (Dalyander et al. 2014). Oil delivered to the beaches forms sediment-oil agglomerates, which undergo further degradation, releasing various intermediate water-soluble organic components (Bociu et al. 2019). These photooxidized products are potentially toxic to organisms thereby increasing the overall toxicity of seawater exposed to the oil (King et al. 2014; Maki et al. 2001). Major crude oil components found in spill-affected waters are alkanes, cycloalkanes, aromatics, polycyclic isoprenoids, resins, and asphaltenes (Ferguson et al. 2020). Among them, the aromatic components, including the polycyclic aromatic hydrocarbons (PAHs), are particularly hazardous due to their environmental persistence and biological toxicity.

Natural seepage of oil hydrocarbons from subsea oil reservoirs is prominent in the Red Sea (Bourtsoukidis et al. 2020), in addition to other significant sources such as oil-related coastal industrial operations and intense marine transport of oil between Asia and the Western Hemisphere in tankers (Periáñez 2020). Moreover, recurrent oil spills of various magnitudes in the northern Red Sea have adversely affected the coastal areas, negatively impacting the environment and economy (Kostianaia et al. 2020). Such oil contamination in the Red Sea is also a rising concern to its biodiversity-rich coastal habitats, such as coral reefs, mangrove forests, seagrass beds, and other soft substrate habitats. This, in turn affects the aesthetic and economic value of the coast and its potential to support tourism. Therefore, oil pollution remains a prominent concern for the Red Sea coastal habitats (Al-Mur 2019).

The Red Sea represents a semi-enclosed ocean basin with a higher sea surface temperature (SST) than other tropical regions, ranging from 22 to > 32 °C (Raitsos et al. 2011). According to several studies, the biodegradability of crude oil can be elevated by warmer temperatures due to an upregulation of oil-degrading microbial metabolism and enzyme activities (Rahman et al. 2002; Rowland et al. 2000; Zekri & Chaalal 2005). Therefore, warm Red Sea waters are expected to accelerate the rate of microbial degradation of oil in the surface layers, particularly that of alkane biodegradation (Campo et al. 2013). Moreover, high incident solar radiation in the region, along with the transparent waters and deep penetration of ultraviolet radiation (UVR) (Overmans & Agustí, 2020) could promote photocatalytic decomposition and photodissolution of hydrocarbons in the surface oil, which are among the widely recognized fate processes in the ocean (Freeman & Ward 2022). In addition, hydrocarbon-degrading bacteria dominate the Red Sea coastal microbiome (Mustafa et al. 2016). Hence, the Red Sea is expected to provide ideal environmental and microbial settings for the rapid natural degradation of oil residues in the surface layers.

Crude oil degradation under realistic field conditions has been demonstrated in the warmer Arabian Gulf (Saeed et al. 2011) and the Gulf of Mexico (Bacosa et al. 2015). However, a knowledge gap exists regarding the dissolution of oil hydrocarbons in the warmer and highly transparent Red Sea and the role of these physical factors. Moreover, studies on oil hydrocarbon cycling and microbial responses associated with oil spills are rare in the Red Sea, despite the high risk of oil contamination in the region due to inadequate sampling and replication during and after oil spill events. Here, we assess the impacts of SST and solar radiation typical of the Red Sea region on the dissolution of crude oil collected on the beach after an oil spill occurred in the Saudi Arabian Red Sea following an explosion of the oil tanker Sabiti (at 38.33°E, 21.13°N) on October 11, 2019 (Nukapothula et al. 2021; Periáñez 2020). The eastern coast of the Saudi Arabian Red Sea, near the port city of Jeddah, experienced significant impact from the released oil (Vankayalapati et al. 2023). We analyzed oil dissolution rates and associated changes in the spectral properties of seawater, signals of fluorescent dissolved organic matter (FDOM), isotopic composition, and resident bacterial community dynamics in water exposed to oil under different solar radiation and temperature regimes. We specifically examined shifts in the stable isotope ratio (δ^13^C) of the dissolved organic matter (DOM) as an indicator of the fate of oil carbon in the seawater through microbial heterotrophic activities and carbon cycling. In addition, post-experiment chemical analyses of the water for aromatic degradation products revealed significant insights into the environmental consequences of photooxidation of crude oil driven to the shores after accidental spills in the open ocean. The results indicated that the interactive effect of temperature and solar radiation contributed to enhancing the dissolution of oil on the Red Sea surface.

## Materials and methods

### Collection and processing of crude oil sample

Four days after the Sabiti tanker oil spill incident on 11 October 2019, heterogeneous coastal sediment sample covered by thick oil agglomerates was hand-collected randomly while wearing sterile nitrile gloves and stored in amber glass jars at the Al-Saif beach along Jeddah coast, Saudi Arabia (39.17°E, 21.16°N; Fig. S1). The sample was stored for 2 days at 4 °C until processing. In the laboratory, large debris particles were manually removed. The concentration of total petroleum hydrocarbons (TPH; C_8_ − C_40_) in the crude sample was measured by gas chromatography coupled with flame ionization detection (GC-FID) scan following the reference method, DEP-FL-PRO-2018 (Supplementary Methods S1), and PAHs were analyzed by gas chromatography tandem triple-quadrupole mass spectrometry (GC–MS/MS, Agilent 7890 GC 7010B/MS) after accelerated solvent extraction (ASE) using dichloromethane as solvent (Supplementary Methods S2).

To refine the crude oil component from the sediment, 100 mL of dichloromethane (DCM) was added to the agglomerate in an approximately 1:2 w/w ratio. After thorough mixing, the DCM fraction with oil was separated using a separating funnel, filtered through a 100 μm mesh, and collected in a wide-mouth amber glass bottle. DCM was allowed to evaporate at room temperature inside a fume hood. After reducing the volume to approximately 20 mL, the extract was sealed in 40 mL amber screw-top vials and stored at 4 °C until use.

### Experimental setup

The experiment was conducted in outdoor incubation tanks at the Coastal and Marine Resources Core Lab (CMOR) at the King Abdullah University of Science and Technology (KAUST), equipped with continuous raw seawater circulation at 26 °C and 30 °C, which represent the mean and summer high values of Red Sea SST, respectively (Chaidez et al. 2017), and under two levels of natural sunlight (Fig. S2). The temperature was controlled within ± 0.5 °C using an underwater thermostat (SCHEGO Schemel & Goetz GmbH & Co) and was monitored daily with a high-precision digital thermometer (Catalog # 89369–138; VWR International, United States). Quartz glass flasks (Multi-Lab, Newcastle, UK; flask height 28 cm; bottom diameter 15 cm; mouth diameter 6.5 cm) which are transparent to the full solar spectrum, containing 2 L of GF/F filtered natural Red Sea seawater, were placed in the tanks. Approximately 1 g of purified crude oil was carefully added to each flask to form thin surface films of approximately 2 mm thickness and 8 cm diameter. The flasks were tightly capped with approximately 10 cm of headspace and partially immersed in the circulating water, ensuring that the seawater with floating crude oil film remained within the desired temperature. An incubation experiment for 4 days was established with the following treatments at two temperatures: seawater control without oil film (SWC), oil film under dark (D), oil under reduced solar radiation (RSR), and oil under full solar radiation (FSR). The RSR was achieved by covering the flasks with a neutral mesh cloth to reduce the irradiance by 50% across the UV and visible spectra while flasks under dark treatment were wrapped in aluminum foil. The irradiance incident at the level of the oil films was continuously monitored using a data-logging radiometer (PMA2100, Solar Light, Glenside, PA, USA). At the end of the experiment, the oil films were physically modified unevenly across the treatments thereby hindering the collection of identical oil samples for analysis. Therefore, fluorescence fingerprinting of oil signals in the water-soluble fraction was opted to analyze the degradation products. At the end of each day, 50 mL water samples were carefully collected below the oil film using a metallic syringe without disturbing the film. An equivalent volume of fresh seawater was not added to the flasks to avoid any dilution effect on the target parameters. The change in test volume was uniform across the various treatments; therefore, its impact on the overall conclusions of our study would be negligible.

### Chromophoric dissolved organic matter (CDOM) spectra

The UV–visible absorption spectra of CDOM were measured using a UV–Vis spectrophotometer (Lambda 1050, PerkinElmer, Waltham, MA, USA). Then, 1 mL seawater samples were filtered through 0.2 μm PTFE membrane filters (Pall Life Sciences, Ann Arbor, MI, USA). The CDOM spectra were recorded immediately after filtration in 10-cm path length quartz cuvettes. The measurement range was 250–750 nm with 1 nm intervals. Filtered fresh Milli-Q water was used as blank (Li et al. 2009). The CDOM spectra were processed according to Iuculano et al. (2019). In brief, the CDOM spectra were adjusted by subtracting the blank spectra to eliminate the majority of the Raman scattering (Liu et al. 2021). All absorbance data points were corrected to the average absorbance from 600 to 750 nm to nullify the residual scattering properties of the sample. Then, the absorbance values were transformed to Naperian absorption coefficient a_*λ*_ (m^−1^) using the equation provided elsewhere (Iuculano et al. 2019). The variability and quality of CDOM were assessed by two proxy parameters, namely, absorption coefficients at 254 nm and 325 nm (a_254_ and a_325_, respectively). a_254_ is proportional to the number of conjugated carbon double bonds and a proxy to the bulk dissolved organic carbon (DOC) concentration, and a_325_ is representative of aromatic CDOM components (Iuculano et al. 2019).

### Fluorescence emission spectra

Fluorescence fingerprinting of FDOM components in seawater was carried out at room temperature with a 1 cm quartz cuvette using a fluorescence spectrometer (LS55, PerkinElmer, Waltham, MA, USA) calibrated with quinine sulfate standard solution (Zhou et al. 2013). For the single exposure detection of fluorescence signatures, excitation-emission pairs at 254/350 nm and 350/410 − 550 nm were used for light/refined and heavy oil components, respectively (Lambert 2003). The variation in FDOM fluorescence was analyzed by generating excitation-emission matrix (EEM) spectra (Zhou et al. 2013). Thirty-six individual fluorescence emission spectra were measured from 240 to 680 nm with a 0.5 nm interval under excitation wavelengths from 220 to 400 nm with a 4 nm step. The scan speed was set at 250 nm min^−1^. All spectra were generated at a constant room temperature of 23 ± 1 °C. The spectrum of Milli-Q water blank was scanned under the same conditions and subtracted from each sample spectrum to eliminate water Raman scatter peaks.

### Total organic carbon (TOC)

Water samples for TOC analysis were collected in acid-cleaned, pre-combusted glass tubes (40 mL), acidified with 85% H_3_PO_4_ (at 0.2% v/v), and kept refrigerated in the dark at 4 °C until analysis. TOC was estimated by high-temperature catalytic oxidation (HTCO) in a Shimadzu Total Organic Carbon Analyzer (TOC-L, Shimadzu Corp., Kyoto, Japan), following the method described previously (Calleja et al. 2019). The instrument was standardized by generating a potassium hydrogen phthalate calibration curve. The accuracy of the measurements was monitored by referencing each sample against Consensus Reference material of deep-sea water (42–45 μmol C L^−1^) and low carbon water (1–2 μmol C L^−1^) allowing a resolution of 1.4 μmol L^−1^ (Calleja et al. 2019).

### PAHs in seawater

The concentrations of 18 different PAHs (16 EPA PAH, 1-methylnapthalene, and 2-methylnapthalene) were analyzed in water samples collected from the experimental flasks at the onset and at the end of the experiment. Water samples from the treatments were filtered to remove the oil fragments, collected in 125 mL amber glass bottles with Teflon™-lined screw-caps, acidified to pH 2, and stored frozen at −20 °C until analysis. Total residual PAHs in water were analyzed by solid-phase extraction (SPE) using an EnvirElut-PAH cartridge (1 g/6 mL, Agilent, Santa Clara, CA), followed by GC–MS/MS (Agilent 7890 GC 7010B/MS) as described in Supplementary Methods S3. The transitions used for PAH determination are provided in Table S1. Our method included specific quality control parameters that are applicable to the US-EPA Method 8270 D.

### Flow cytometric determination of bacterial abundance

Microbial activity in response to oil dissolution was monitored by counting the total heterotrophic bacteria abundance, discriminating between high nucleic acid (HNA) and low nucleic acid (LNA) bacteria groups using flow cytometry. At the daily sampling event, 2 mL subsamples were collected in cryo-vials and fixed with 80 µL of glutaraldehyde (25% v/v). Samples were flash-frozen in liquid nitrogen and stored at −80 °C. All samples were analyzed within 2 weeks of collection. Bacterial abundance was estimated using the BD FACSCanto II flow cytometer (BD Biosciences, Eysins, Switzerland) as described in Supplementary Methods S4.

### CH_4_, CO_2_, and isotopic composition (δ.^13^C)

The concentrations and stable carbon isotopic composition of CH_4_ (δ^13^C-CH_4_) and CO_2_ (δ^13^C-CO_2_) were measured at the end of the experiments by headspace-cavity ring-down spectroscopy (CM-CRDS G2201-I, Picarro Inc, Santa Clara CA, USA) following (Sea et al. 2018). At the end of incubation, 1 L of water from each flask was transferred to clean glass bottles and equilibrated for 1 h between the seawater and the headspace air. 10 mL of the headspace air was subsequently sampled in a syringe and injected into the spectrometer using a Small Sample Isotopic Module extension (SSIM A0314, Picarro Inc., Santa Clara, CA, USA), to determine the partial pressure and the isotopic carbon composition of the CO_2_ and CH_4_ in the air sample (Burkholz et al. 2020). The standard used was an industrial air mixture (750 ppm CO_2_ and 9.7 ppm CH_4_; Abdullah Hashim Industrial Gases & Equipment Co. Ltd., Jeddah, Saudi Arabia). The CRDS system measures methane levels as low as 0.1 ppm. The analytical precision of δ^13^C*-*CH_4_ and δ^13^C*-*CO_2_ measurements was ± 1.5 ‰ and ± 0.2 ‰, respectively.

### Statistical analyses

The rate of change in TOC was obtained as the slope of the TOC-time relationship. The total carbon in the initial oil film was estimated to be 471 mg (Table S2). Assuming that the change in TOC in the seawater is oil-derived, the corresponding change in C content in the film was empirically calculated. Based on the changes in C content, the dissolution rate of crude oil film (*R*, g C m^−3^ d^−1^) was calculated as

1 $$R=1-\left(\frac{{C}_{2}}{{C}_{1}}\right)\times 100$$

where *R* is the dissolution rate, *C*_2_ is the final C content, and *C*_1_ is the initial C content of the oil film.

All results were tested for treatment effects on various parameters using two-way analysis of variance (ANOVA) with temperature and light conditions as main factors, and their interactions, at a significance level of 0.05, using JMP software (JMP Pro version 13.1, SAS Institute, USA). The EEM spectra were analyzed and contour plots were generated using Origin software (Origin-Lab, Northampton, MA, USA). Nonparametric Spearman’s rank correlation test was performed to show the relationship between different variables measured in seawater after oil amendment (JMP software). Correlations (Spearman’s correlation coefficient (*ρ*)) were considered statistically significant when *p* < 0.05.

## Results

### Changes in PAHs, dissolved carbon concentration, and associated optical properties

Our FSR treatment represents exposure to an average daily maximum incident solar photosynthetically active radiation (PAR) and UVR at 910 ± 206 µE m^−2^s^−1^ and 123 ± 27 W m^−2^, respectively (Fig. S3 a, b). The original crude oil sample recorded a sum of 146,580 mg kg^−1^ TPHs, with total carbon and hydrogen levels of 47.1% and 9.1%, respectively (Table S2). PAHs in the crude oil sample were dominated by 3 − 4 ringed compounds. However, the background PAHs in the experimental seawater were negligible, with the detection of the two PAHs, fluoranthene (0.08 μg l^−1^) and phenanthrene (0.13 μg l^−1^). After the incubation with an oil film, these values increased up to 0.1 μg l^−1^ (FSR at 30 °C) and 0.62 μg l^−1^ (RSR at 30 °C), for fluoranthene and phenanthrene, respectively. In addition, the three-ringed fluorene was detected in the oil-amended seawater within a range of 0.11 − 0.33 μg l^−1^ among treatments, with the highest concentration in the sample incubated at 26 °C under full solar radiation (Table S2).

**Fig. 1:**
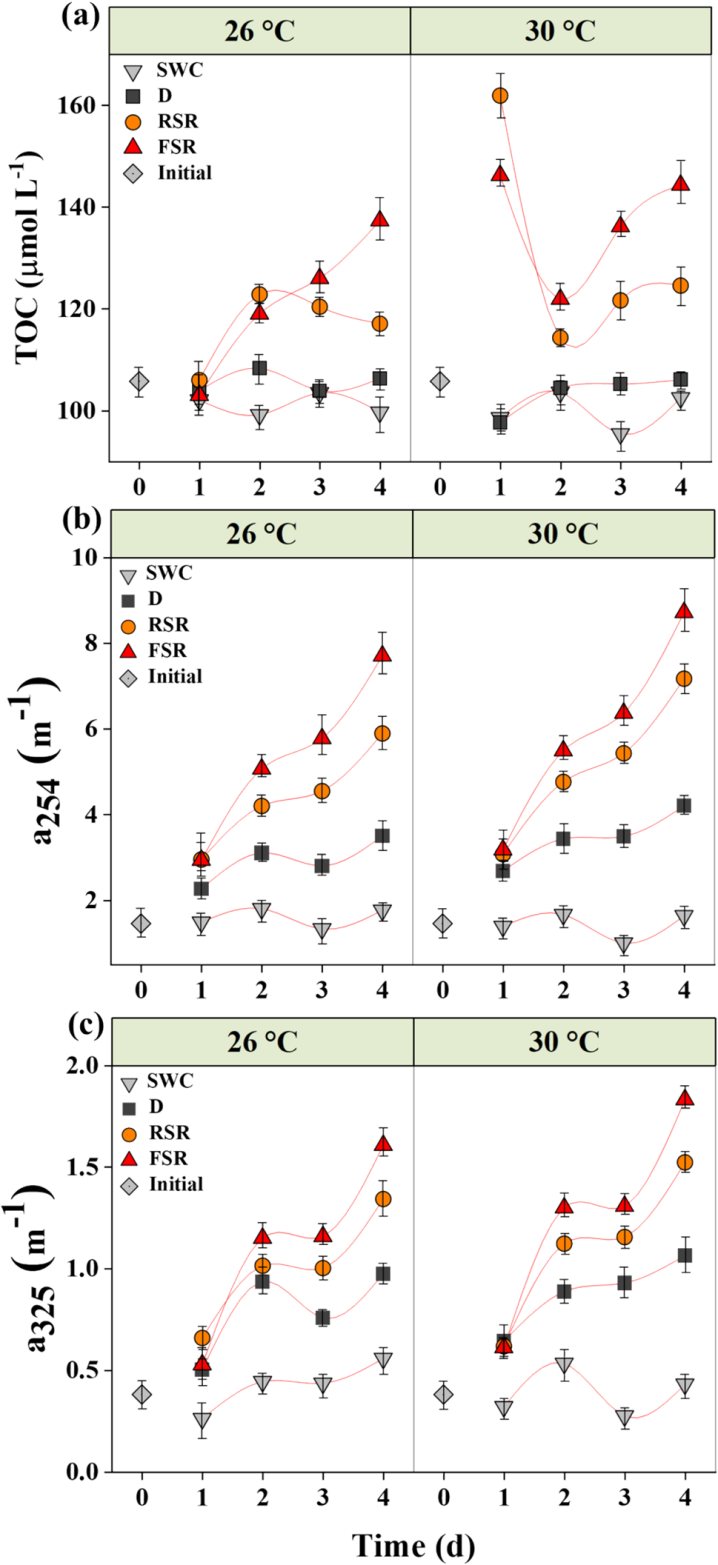
Daily variation in TOC (**a**), CDOM absorption coefficients a_254_ (**b**), and a_325_ (**c**) in seawater incubated with or without crude oil film for 4 days. Abbreviations: SWC, seawater control without oil film under full solar radiation; D, with oil film in the dark; RSR, with oil film under 50% of solar radiation; FSR, with oil film under full solar radiation

The initial TOC concentration in experimental seawater, before adding the oil, was 106 ± 1.3 μmol C L^−1^. The TOC concentration showed a sharp increase in the oil-amended seawater exposed to sunlight (Fig. 1a). An abrupt rise in the TOC at 30 °C was clearly distinguished within 1 day of incubation, with up to 53% and 38% increase in the TOC concentration under 50% reduced and full incident levels of solar irradiation, respectively, with a much smaller shift in samples incubated at 26 °C (Fig. 1a). However, no significant temporal variation in TOC concentration was observed in the absence of sunlight across treatments. The average TOC in the seawater control samples remained in the range of 98.56 − 103.58 μmol C L^−1^ (Fig. 1a). Significant effects of temperature and solar radiation on changes in the TOC were observed (two-way ANOVA, *p* < 0.01, Table S3).

The dissolution rates of oil films ranged from 0.5 to 38.68 g C m^−3^ d^−1^ and 3.7 to 30.11 g C m^−3^ d^−1^ in 26 °C and 30 °C treatments, respectively (Table 1). Moreover, full solar radiation significantly enhanced the dissolution rates (two-way ANOVA, *p* < 0.001), with dissolution rates 10 to 60 times greater under full solar radiation and 7 to 30 times greater under 50% of incident solar radiation than samples incubated in the dark, depending on temperature (Tables S3 and 1). The highest dissolution rate was recorded in samples exposed to full solar radiation incubated at 26 °C (Table 1). Two-way ANOVA showed a significant interactive effect of temperature and light levels on the dissolution rate of the surface oil films (two-way ANOVA, *p* < 0.001, Table S3).

**Table 1:** Comparison of slopes of total organic carbon (TOC)-time relationship and corresponding crude oil dissolution rates in Red Sea water across various temperature and solar radiation treatments

Treatment	Temperature (± 0.5 °C)	Slope of TOC-time relationship ± SE	Oil dissolution rate (g C m^−3^ d^−1^)
Seawater control (SWC)	26	− 0.013 ± 0.01	− 4.83
	30	− 0.012 ± 0.02	− 4.34
Dark (D)	26	0.001 ± 0.01	0.52
	30	0.01 ± 0.01	3.70
Reduced solar radiation (RSR)	26	0.045 ± 0.02	16.65
	30	0.059 ± 0.01	21.93
Full solar radiation (FSR)	26	0.103 ± 0.02	38.68
	30	0.08 ± 0.06	30.11

The CDOM absorption coefficients a_254_ and a_325_ significantly increased in the treatments amended with oil compared to seawater controls at the two temperatures tested (Fig. 1b, c). The a_254_ and a_325_ absorption coefficients increased from initial values of 1.45 ± 0.02 m^−1^ and 0.38 ± 0.01 m^−1^, respectively, to 8.71 ± 0.05 m^−1^ and 1.83 ± 0.01 m^−1^, respectively, under full solar radiation at 30 °C. The rise in a_254_ and a_325_ was significantly higher at 30 °C and under full solar radiation at both temperatures (Fig. 1b, c). The increase in DOM absorption was significantly influenced by high solar radiation (2-way ANOVA, *p* = 0.001, Table S3), but not temperature (*p* = 0.19).

**Fig. 2:**
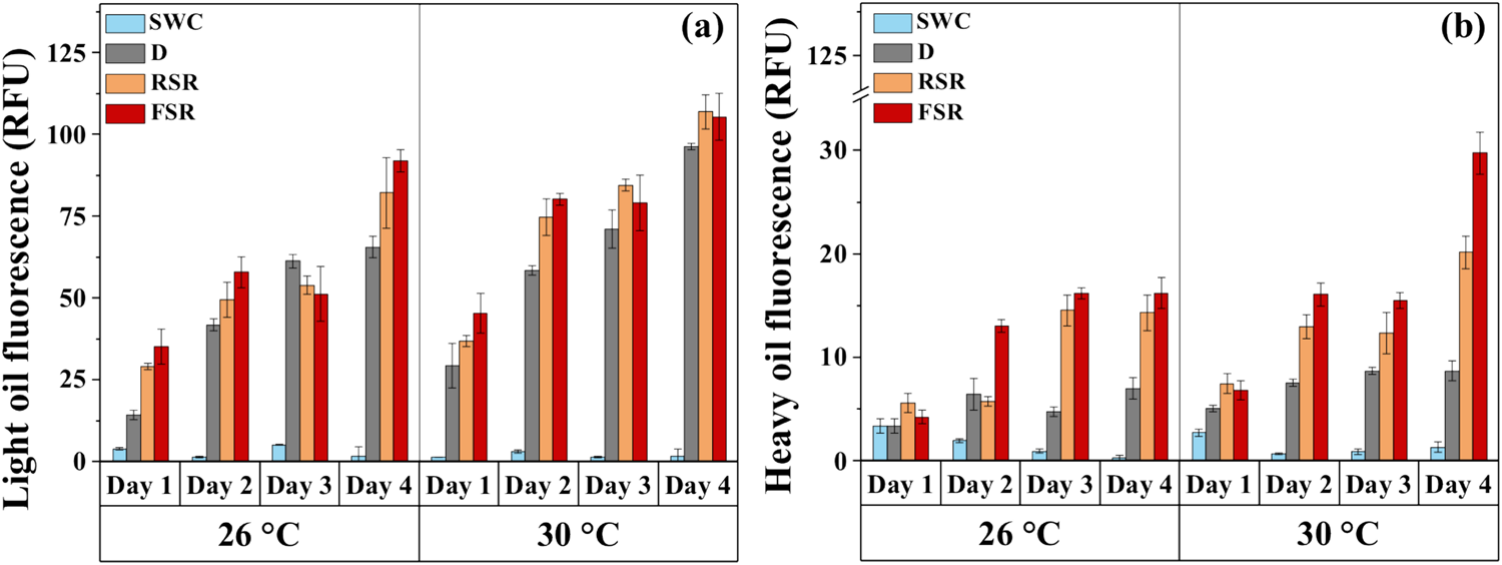
Daily variation in fluorescence emission of light (**a**) and heavy (**b**) oil components in seawater during incubation with or without crude oil films. Abbreviations: SWC, seawater control without oil film under full solar radiation; D, with oil film in the dark; RSR, with oil film under 50% of solar radiation; FSR, with oil film under full solar radiation

The FDOM fluorescence spectra showed a significant increase in the emission of light and heavy components throughout the experiment in all oil-amended treatments. On day 4, the emission at 350 nm ranged between 66 and 92 relative fluorescence units (RFU) compared to 1.75 RFU in the seawater control (Fig. 2a). The light oil fluorescence emission was statistically different between various oil treatments (Student’s *t*-test, *p* < 0.05) at both temperatures. Two-way ANOVA revealed significant effects of temperature (*p* < 0.001, Table S3) and solar radiation (*p* = 0.03, Table S3) on 4 days amendment of light oil fluorescence signals in seawater, along with a significant influence of temperature in the absence of sunlight (Student’s *t*-test, *p* < 0.05). However, the interactive effect of temperature and solar radiation was not significant (two-way ANOVA, *p* = 0.38, Table S3).

The heavy oil fraction, as indicated by the emission at 450 nm (Fig. 2b), was relatively lower in magnitude than that of the light oils, indicated by the emission at 350 nm. The heavy oil fluorescence signal showed the lowest values in the controls by the end of the experiment (0.08 RFU and 1.35 RFU at 26 °C and 30 °C, respectively). However, under full solar radiation, it increased to 16 RFU and 30 RFU at 26 °C and 30 °C, respectively, showing significant single and combined effects of temperature and solar radiation (2-way ANOVA, *p* < 0.05, Table S3). Moreover, the values at 30 °C were significantly higher than those at 26 °C at both irradiance levels.

The area under the integrated light oil fluorescence curve (Ex 254 nm, Em 250 − 650 nm) at the end of incubation varied significantly among treatments, with 21% and 24% increase at 26 °C and 30 °C, respectively, under full solar radiation (Fig. S4). The results from ANOVA indicated that high solar radiation significantly enhanced (DF = 2, *F* = 501.38, *p* < 0.0001) the integrated area of the fluorescence curves, along with a significant effect of temperature (DF = 2, *F* = 248.12, *p* < 0.0001), but no significant interaction between temperature and solar radiation (2-way ANOVA, DF = 2, *F* = 0.52, *p* = 0.61, Table S3).

**Fig. 3:**
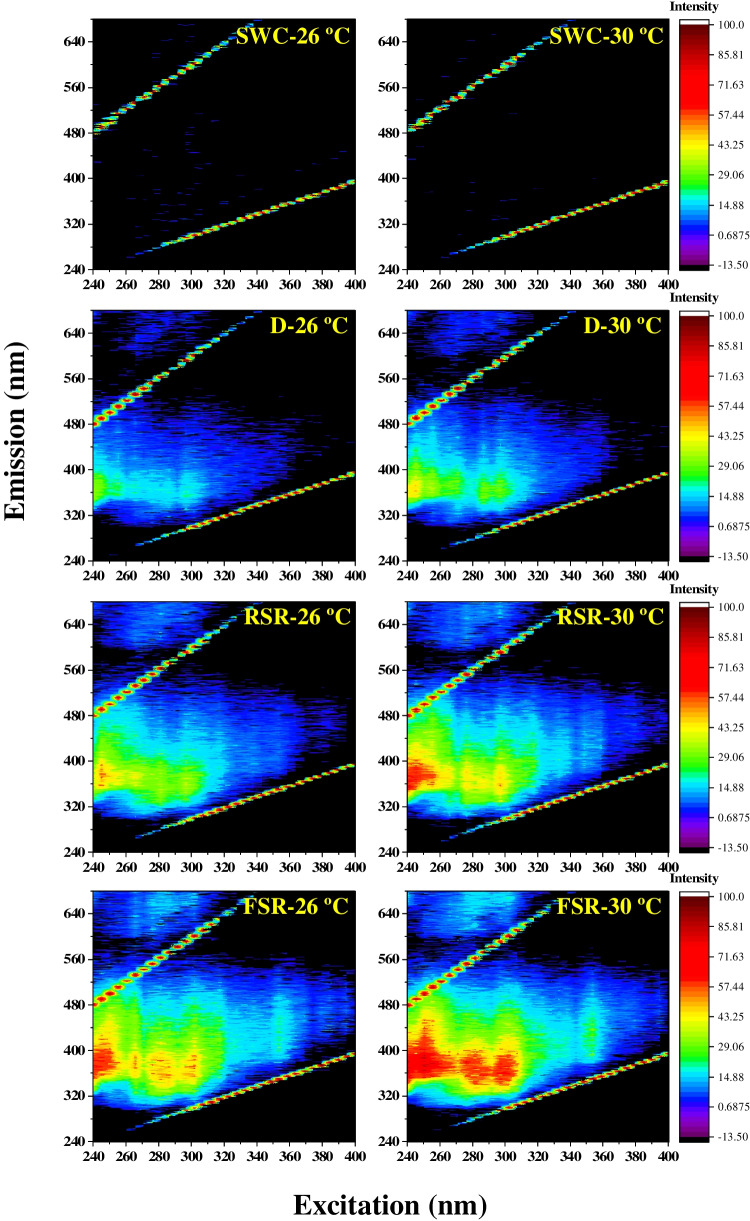
Excitation-emission matrix (EEM) contour plots generated on day 4 for Red Sea water incubated with or without crude oil at 26 °C and 30 °C. Abbreviations: SWC, seawater control without oil film under full solar radiation; D, with oil film in the dark; RSR, with oil film under 50% of solar radiation; FSR, with oil film under full solar radiation

The contour plots of normalized excitation-emission matrix spectra (EEMs) clearly showed the amendment of seawater with oil-derived fluorophores and the influence of solar radiation and temperature (Fig. 3). All the oil-amended treatments showed distinct fluorescence emission bands in the 340–420 nm range over an excitation range of 240–310 nm. In the samples receiving the full incident solar radiation, multiple bands were clearly distinguished within this range. Three major emission bands centered at about 370 nm appeared with distinct excitation maxima at 250 nm, 280 nm, and 300 nm (Fig. 3). In the treatment incubated at 26 °C under full solar radiation, the component with Ex/Em maximum pair at 245/380 nm was the dominant fraction in samples amended with an oil film. However, samples incubated at 30 °C showed additional major components with Ex/Em maxima pairs at 280/370 nm and 300/370 nm (Fig. 3). Samples incubated at full solar radiation and 30 °C showed an additional signal with Ex/Em maxima at 250/440 nm.

### Shifts in bacterial community and growth rates

Microcosm incubation of oil-amended seawater resulted in significant shifts in bacterial community structure from the untreated controls. The initial proportions of the LNA and HNA groups were 71% and 29%, respectively. However, the relative abundance of the HNA population increased significantly in all the treatments (Fig. S5). In seawater exposed to oil under sunlight, a higher proportion of the HNA population was observed, rising to comprise 70 − 90% of the total community. On the other hand, the LNA population drastically declined in all treatments. The reduction was more severe after the oil amendment under 30 °C, where LNA declined to < 10% by the end of the experiment (Fig. S5 b).

The HNA bacteria responded to oil dissolution by showing a higher average cellular growth rate in treatments under sunlight (0.58 ± 0.03 day^−1^) than those incubated in the dark (0.40 ± 0.02 day^−1^), which showed a similar growth rate as seawater control (0.43 ± 0.03 day^−1^). Two-way ANOVA analysis indicated that the HNA growth rate significantly varied with temperature and solar radiation, including a significant positive interaction between these two (DF = 2, *F* = 27.83, *p* < 0.0001, Table S3). However, the LNA growth rates significantly declined from 0.26 ± 0.01 day^−1^ in the seawater controls to 0.19 ± 0.01 day^−1^ in the dark, further showing a mean growth rate of − 0.08 ± 0.02 day^−1^, indicative of decay, in the treatments with oil under sunlight (Student’s *t*-test, *p* < 0.05).

### CO_2_ and CH_4_ levels in the equilibrated air and their isotopic composition (δ.^13^C)

The concentration of CH_4_ significantly decreased from an initial concentration of 1.79 ppm to 0.42 ppm in the FSR treatment at 26 °C at the end of the incubation (Table 2). However, CH_4_ levels increased in the dark treatments. At the end of the incubation, CH_4_ was below the detection limit in the FSR treatment at 30 °C. Two-way ANOVA revealed significant interactive effects of temperature and solar radiation on the headspace CH_4_ concentrations (DF = 2, *F* = 92.49, *p* < 0.0001, Table S3). The headspace CO_2_ concentrations significantly increased in the presence of oil at 30 °C. However, there were no significant changes in CO_2_ at 26 °C, except for an 11% increase in the dark treatment.

**Table 2:** Comparison of CO_2_ and CH_4_ levels and their δ^13^C values in Red Sea water after various incubations with surface oil film for 4 days. *ND*: not detected

Treatment	Temperature (± 0.5 °C)	CO_2_ ± SE (ppm)	CH_4_ ± SE (ppm)	δ^13^C-CO_2_ ± SE (‰)	δ^13^C-CH_4_ ± SE (‰)
Seawater control (SWC)	26	361 ± 28	1.57 ± 0.05	− 15.01 ± 0.9	− 19.74 ± 4.1
	30	343 ± 6	1.47 ± 0.04	− 18.73 ± 1.2	− 14.07 ± 1.6
Dark	26	402 ± 4	1.87 ± 0.03	− 12.93 ± 0.2	− 46.61 ± 1.7
	30	371 ± 4	4.15 ± 0.07	− 10.40 ± 0.4	− 8.09 ± 1.1
Reduced solar radiation (RSR)	26	368 ± 3	1.42 ± 0.03	− 20.13 ± 0.5	− 29.27 ± 5.2
	30	367 ± 7	1.31 ± 0.04	− 22.61 ± 0.9	− 45.89 ± 6.9
Full solar radiation (FSR)	26	374 ± 4	0.42 ± 0.45	− 31.24 ± 3.5	ND
	30	359 ± 1	ND	− 44.51 ± 2.6	ND

The mean isotopic signature of δ^13^C-CH_4_ was significantly heavier in the oil-amended flask exposed to low solar radiation (− 29.27 and − 45.89‰ at 26 °C and 30 °C, respectively) than in the seawater controls (− 19.74 and − 14.07‰ at 26 °C and 30 °C, respectively). However, the δ^13^C-CO_2_ values were more negative in oil-amended treatments (ranging from − 19.62 to − 47.08‰) than that in the seawater controls (ranging from − 14.06 to − 19.95‰, Table 2). Moreover, a significant combined effect of temperature and light level on the shift in δ^13^C-CO_2_ was observed (two-way ANOVA, *p* < 0.0001, Table S3).

## Discussion

### Impacts of photodissolution of oil hydrocarbons on seawater properties

Oil agglomerates and sludges deposited on shores indicate crude petroleum pollution in coastal areas after accidental spills in the oceans (Bociu et al. 2019; Gustitus & Clement 2017). Beach-stranded oil is detrimental to the coastal environment due to the presence of toxic components such as oxygenated hydrocarbons, free radicals, and persistent PAHs (John et al. 2016). The oil sample used in this study was collected 4 days after it was released from the tanker, floated on the sea surface, and deposited in the coastal region of the Red Sea. Therefore, the oil was exposed to substantial natural weathering and dissolution processes prior to sample collection. Nevertheless, tidal currents and waves carry the soluble degradation products from oiled shorelines to the seawater resulting in chronic contamination and biological adverse effects (Gustitus & Clement 2017; Kim et al. 2013).

Natural sunlight is a strong factor enhancing crude oil degradation through generation of superoxide radicals (Yan et al. 2023) and changes in bacterial community structure (Bacosa et al. 2015). A major pathway involved in sunlight-driven oil transformation is the formation highly polar oxygenated compounds from petroleum which results in higher partitioning to the oil–water interphase (Zito et al. 2020). Natural sunlight particularly accelerates degradation of large, biorefractory PAHs which releases toxic, water-soluble photoproducts (King et al. 2014). The variation in TOC and CDOM in our study clearly indicates that solar radiation alters biogeochemical cycling of petroleum hydrocarbons entering the aqueous phase from surface films.

Photodissolution and biodegradation are the key weathering processes of crude oil in the surface waters. The fate of spilled oil in the ocean is controlled by complex interplays between crude oil characteristics and environmental parameters such as salinity, temperature, and exposure to light. Specifically, oceanic oil spills trigger microbial community dynamics depending on the quality of the spilled oil and the environmental conditions at the affected site (Boetius 2011). Moreover, the amount of carbon increases during an oil spill, and availability of nutrients (nitrogen and phosphorus) influences the microbial processes leading to biodegradation of the oil (Leahy & Colwell 1990). Natural sunlight is a predominant driver of oil dissolution, particularly for thick floating slicks (Freeman & Ward 2022). We recorded higher dissolution rates of surface crude oil (> 30 g C m^−3^ d^−1^) than values (0.01 − 0.3 g C m^−3^ d^−1^) reported in the previous field and mesocosm studies (Stewart et al. 1993). Temperature and sunlight are critical factors and their combined effect can alter the physicochemical properties of the oil and oil-degrading marine microbial community dynamics, thereby affecting the rates of photooxidation (Saeed et al. 2011), biodegradation (Bacosa et al. 2015; Neethu et al. 2019), and various physical degradation processes (John et al. 2016). Thus, the high temperature and sunlight prevalent in the Red Sea can significantly influence the weathering of crude oil, as reported in the warmer surface waters (28–30 °C in summer) of the Gulf of Mexico (Bacosa et al. 2018). The Red Sea also has remarkably low levels of oil residues in the water phase (Al-Lihaibi 2003) despite high effluent input and extensive oil-related activities in the area, due to the presence of diverse hydrocarbon-degrading microbial communities (Bargiela et al. 2015; Hanafy et al. 2016). Although a significant conjoint effect of temperature and sunlight was evident on the water-soluble oil components, the dissolution rate in our FSR treatment was greater at 26 °C, than at 30 °C, which may be due to the rapid saturation of microbial activities at higher temperature.

Microcosm exposure of oil films to natural sunlight and temperature equivalent to those in the Red Sea (Overmans & Agustí, 2020; Raitsos et al. 2011) led to significant oil dissolution and subsequent rapid qualitative changes in complex hydrocarbon mixtures in the seawater. Variation in TOC and CDOM in the water clearly indicated that the dissolution of oil hydrocarbons contributed to important constituents of the DOM. In an open ocean oil spill scenario, the rates of oil dissolution and the release of photooxidation products into the seawater are influenced by physical factors such as latitudinal and seasonal variation in solar irradiance (Freeman & Ward 2022), temperature, UV irradiance, and oxygen levels (Saeed et al. 2011). In general, the dissolved PAHs in seawater were dominated by low molecular weight (LMW) compounds with high solubility. The background levels of PAHs in the Red Sea waters were negligible (Table S2), possibly helped by effective hydrocarbon-degrading microorganisms (Röthig et al. 2017; Shetaia et al. 2016) which significantly attenuate PAHs in the dissolved phase. Moreover, natural sunlight acts as a key factor in stimulating the abundance of oil-degrading phylotypes of bacteria (Bacosa et al. 2015).

TOC in the experimental seawater was significantly higher than the baseline value of 66 − 100 μmol l^−1^ reported in the Saudi Arabian coast of the Red Sea (Dehwah et al. 2015). The soluble carbon from the oil film resulted in a slight increase in TOC and CDOM absorbance in the dark (Fig. 1a, b). However, the rapid increase in TOC during the initial hours of sunlight incubation indicates the physical dissolution of organic components, where photooxidation enhanced their aqueous solubility (Ray et al. 2014). A remarkable change in TOC and CDOM on the third day of incubation (Fig. 1a, b) can also be attributed to active bacterial metabolism. The slope of the CDOM absorption spectrum is inversely proportional to the molecular weight of DOM components (Helms et al. 2008). Therefore, an increase in CDOM in the oil-amended seawater during the first 2 days of incubation can be attributed to short-term weathering processes that occur at a faster rate in the early hours of the incubation (John et al. 2016; Li et al. 2009) which release water-soluble degradation products from the oil films. However, the soluble organics incorporated into the DOM pool would be subjected to further oxygenation and degradation in the microcosm. For example, high CDOM absorbance and FDOM fluorescence emission were observed in the dark, contrary to steady TOC levels. This implies the modification of DOM components to fluorophores and chromophores under the dark, mediated by microbial activities.

TOC in the experimental seawater was significantly higher than the baseline value of 66 − 100 μmol l^−1^ reported in the Saudi Arabian coast of the Red Sea (Dehwah et al. 2015). The soluble carbon from the oil film resulted in a slight increase in TOC and CDOM absorbance in the dark (Fig. 1a, b). However, the rapid increase in TOC during the initial hours of sunlight incubation indicates the physical dissolution of organic components, where photooxidation enhanced their aqueous solubility (Ray et al. 2014). A remarkable change in TOC and CDOM on the third day of incubation (Fig. 1a, b) can also be attributed to active bacterial metabolism. The slope of the CDOM absorption spectrum is inversely proportional to the molecular weight of DOM components (Helms et al. 2008). Therefore, an increase in CDOM in the oil-amended seawater during the first 2 days of incubation can be attributed to short-term weathering processes that occur at a faster rate in the early hours of the incubation (John et al. 2016; Li et al. 2009) which release water-soluble degradation products from the oil films. However, the soluble organics incorporated into the DOM pool would be subjected to further oxygenation and degradation in the microcosm. For example, high CDOM absorbance and FDOM fluorescence emission were observed in the dark, contrary to steady TOC levels. This implies the modification of DOM components to fluorophores and chromophores under the dark, mediated by microbial activities.

TOC in the experimental seawater was significantly higher than the baseline value of 66 − 100 μmol l^−1^ reported in the Saudi Arabian coast of the Red Sea (Dehwah et al. 2015). The soluble carbon from the oil film resulted in a slight increase in TOC and CDOM absorbance in the dark (Fig. 1a, b). However, the rapid increase in TOC during the initial hours of sunlight incubation indicates the physical dissolution of organic components, where photooxidation enhanced their aqueous solubility (Ray et al. 2014). A remarkable change in TOC and CDOM on the third day of incubation (Fig. 1a, b) can also be attributed to active bacterial metabolism. The slope of the CDOM absorption spectrum is inversely proportional to the molecular weight of DOM components (Helms et al. 2008). Therefore, an increase in CDOM in the oil-amended seawater during the first 2 days of incubation can be attributed to short-term weathering processes that occur at a faster rate in the early hours of the incubation (John et al. 2016; Li et al. 2009) which release water-soluble degradation products from the oil films. However, the soluble organics incorporated into the DOM pool would be subjected to further oxygenation and degradation in the microcosm. For example, high CDOM absorbance and FDOM fluorescence emission were observed in the dark, contrary to steady TOC levels. This implies the modification of DOM components to fluorophores and chromophores under the dark, mediated by microbial activities.

Rapid analytical techniques for oil detection are necessary components of oil spill response framework in a scientific and environmental perspective (Li et al. 2016). Fluorescence and UV-absorbance spectroscopy are semi-quantitative complementary methods to the conventional quantitative fingerprinting based on GC-FID and GC–MS to explore the composition of DOM in seawater and the rapid fingerprinting of oil-contaminated marine areas with high maritime traffic (Mirnaghi et al. 2018; Zhou et al. 2013). The initial weathering of light components in the oil film releases aromatic hydrocarbons which are optically active compounds with unique fluorescence fingerprints. Since the fluorescence profile of petroleum compounds is related to the electronic bonds in the aromatic compounds therein, the EEM spectra (Fig. 3) correspond to the aromatics profile of the contaminated seawater. In addition, the EEM contour plots revealed the significant impact of temperature and sunlight on oil dissolution. The exposure to high temperature and sunlight enhanced the fluorescence peak with an Ex/Em maximum at 250/440 nm, which likely represents a mixture of UV humic-like DOM originating from microbial degradation of organic matter and the weathered oil (Zhou et al. 2013). The fluorescence excitation peaks correspond to the UV absorbance maxima, indicating the relationship between dominant fluorescent components and a_254_. Moreover, the dominant fluorescence peaks in the FSR treatment (Fig. 3) correspond to the fluorescence maxima of PAHs such as phenanthrene, fluoranthene, and fluorene (Smith et al. 2014). The optical properties of PAHs and their oxidation products are reliable markers of their degradation pathways. Thus, UV radiation and high temperature could have increased not only the volatilization of LMW PAHs but also the photodegradation of high molecular weight (HMW) hydrocarbons that are comparatively less volatile (John et al. 2016).

### Bacterial dynamics and its implications on crude oil dissolution

Our data suggest that oil photodegradation alters the dissolved carbon turnover in the contaminated water, and the bacterial processes play a significant role. Photodissolution of oil and subsequent DOM enrichment in the ocean can stimulate quick responses of heterotrophic microbial activity (Harriman et al. 2017; Redmond & Valentine 2012) and microbial utilization of hydrocarbons (Dubinsky et al. 2013; Neethu et al. 2019). Marine heterotrophic bacteria can respond to organic carbon enrichment with a selective proliferation of active cells showing higher DNA content (Morán et al. 2020) and exposure to sunlight will alleviate this change by enhancing the DOM availability to bacteria (Lindell et al. 1995). We observed a prominent shift in the bacterial population from dormant LNA cells to more active HNA cells and significant correlations (Fig. S6) of HNA growth rates with changes in CDOM (Spearman’s *ρ* = 0.52; *p* < 0.05) and TOC (Spearman’s *ρ* = 0.55; *p* < 0.05), indicating that the dissolution of DOM from the oil film is the key factor regulating microbial growth. Interestingly, the conjoint effect of temperature and solar radiation on HNA bacteria growth rate was consistent with those on CDOM and TOC (two-way ANOVA, *p* < 0.001, Table S3).

Oil spills release methane into the ocean (Dubinsky et al. 2013; Yvon-Lewis et al. 2011), a significant fraction of which can further undergo bacterial oxidation (Valentine et al. 2010). Methane dissolution can also promote methanotrophic bacterial metabolism, the extent of which determines the amount of CH_4_ oxidized to the dissolved inorganic carbon (DIC) pool (Redmond & Valentine 2012). The lower headspace CH_4_ and the significant ^13^C-CH_4_ enrichment in our sunlight-exposed microcosms indicate the prevalence of microbial methanogenic biodegradation in the oil-contaminated water (Berdugo-Clavijo & Gieg 2014; Dubinsky et al. 2013).

The detection of methylnaphthalene isomers (2-methylnaphthalene and 1-methylnaphthalene) in the seawater at FSR treatment at 26 °C (Table S2) demonstrates the bacterial degradation of the aromatic fraction of the oil. Methylnaphthalenes represent the rapidly dissolving oil hydrocarbons and the ratio of 2-methylnaphthalene over 1-methylnaphthalene (2-MN/1-MN) signifies the biodegradation of light aromatics (Gutierrez et al. 2018; Kristensen et al. 2021). The detection of these methylnaphthalene isomers in the FSR treatment at 26 °C (Table S2) and a 2-MN/1-MN ratio of 1.25 demonstrates the bacterial degradation of the aromatic fraction of the oil (Gutierrez et al. 2018; Padrós et al. 1999). The concentrations of these isomers were below detection limits (0.04 μg L^−1^) in the other treatments, hindering a comparison of MN/1-MN ratio across various treatments.

**Fig. 4:**
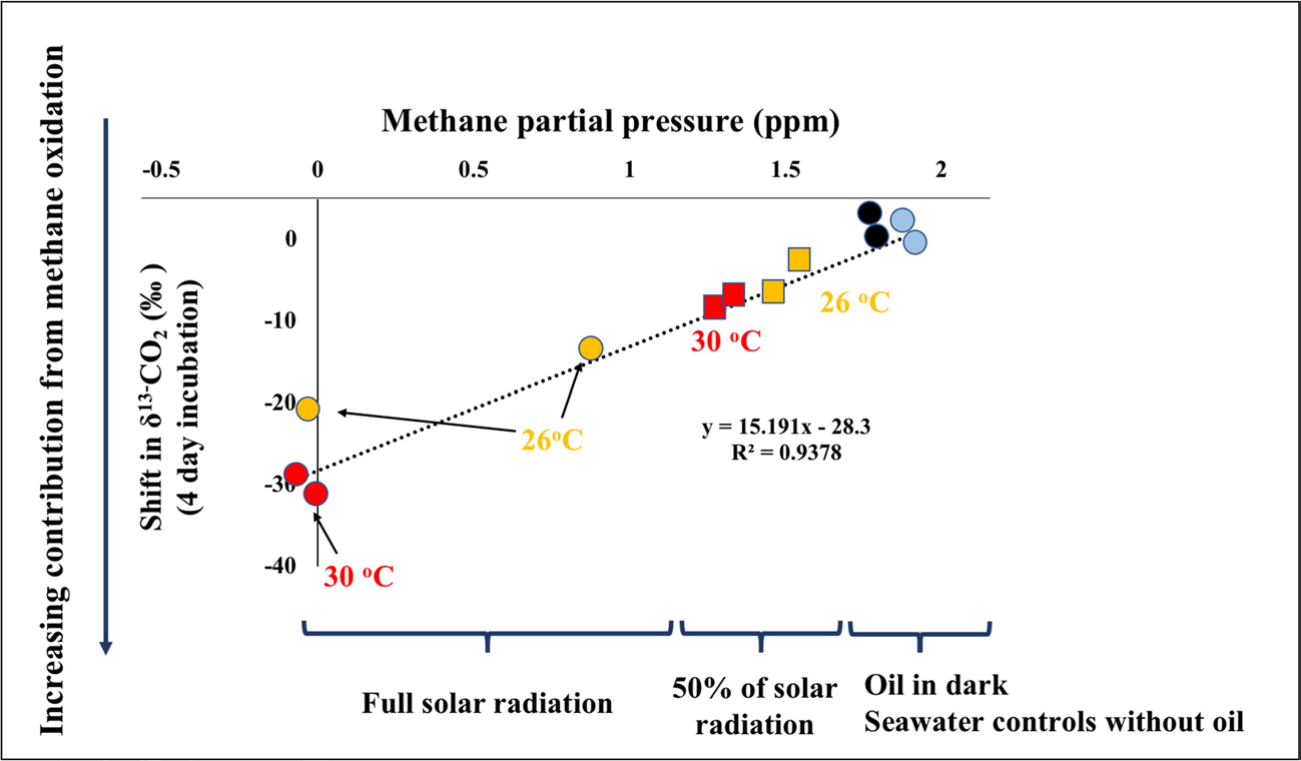
The relationship between CH_4_ partial pressure and variation in δ^13^C-CO_2_ in seawater after various treatments with crude oil films for 4 days. The primary effects of solar radiation and secondary effects of temperature on the overall process are graphically shown. Yellow markers, 26 °C; red markers, 30 °C; black markers, dark incubations; blue markers, seawater controls; squares, reduced solar radiation, circles, full solar radiation

We analyzed the shifts in stable carbon isotope ratio (δ^13^C) of CH_4_ and CO_2_ as an indicator of the rapid movement of oil carbon in the oil-amended seawater through microbial heterotrophic activities and carbon cycling. The δ^13^C of untreated seawater was significantly different between 26 and 30 °C probably due to varying microbial activities and consequent regulation of carbon isotope fractionation at two temperatures. Being a natural product depleted in ^13^C, crude oil has a strongly negative δ^13^C (Medina-Bellver et al. 2005). Therefore, microbial oxidation of hydrocarbons in the surface oil film produces δ^13^C-depleted CO_2_, leading to a decline in the δ^13^C-DIC and a ^13^C enrichment of the residual methane (Dubinsky et al. 2013). Thus, the observed depletion of δ^13^C in CO_2_ reflects the incorporation of oil-derived carbon into the DIC pool through bacterial processes (Fig. 4). The prokaryotic consumers help the movement of carbon from oil and methane to the marine food web, leading to trophic transfer of isotopic carbon depletion and sublethal effects in mesopelagic organisms (Quintana-Rizzo et al. 2015). As stated earlier, the dissolution of oil was significantly enhanced by solar radiation, particularly by UV radiation (Bacosa et al. 2015; Freeman & Ward 2022), where bacterial activities within the oil–water interface release CH_4_, subsequently respired by bacteria and oxidized to CO_2_. A comprehensive analysis of the isotopic data revealed a major influence of solar radiation and a secondary effect of higher temperature on the overall oil dissolution processes (Fig. 4). Moreover, the HNA bacterial response possibly includes a selective proliferation of potential oil-degrading clades which can promptly respond to degradable petroleum after accidental oil spills (Bargiela et al. 2015). Crude oil-degrading bacterial genera (*Pseudomonas*, *Marinobacter*, *Alcanivorax*, *Sphingomonas*, and *Nitratireductor*) predominantly constitute the coastal microbiome in various locations in the Red Sea, as an evolutionary adaptation in response to naturally high background levels of oil hydrocarbons (Hanafy et al. 2016; Mustafa et al. 2016). Consequently, while physicochemical processes result in the transfer and dilution of oil hydrocarbons, biodegradation, in the long term, ultimately removes them from the environment (Nordam et al. 2020).

Shifts in marine DOC levels and fluorescence signals driven by photooxidation of crude oil can persist longer in the water column after oil spills (D’Sa et al. 2016). The results of this study demonstrate that the long-term impacts of oil spills in the Red Sea will likely be affected by the combined effects of climatic and biotic factors. Analysis of individual components of the oil would have provided deeper insights into the effect of high temperature and sunlight on oil dissolution rates; however, this limitation is largely balanced by the relevance and rapidity of information generated by fluorometric and isotopic methods.

This study offers significant insights into crude oil dissolution rates under natural conditions in the Red Sea. However, the generalizability of our findings may be constrained by the limited sample size used in this investigation. Our experimental design did not fully consider potential confounding variables present in the natural environment, including variations in water chemistry, microbial composition, and other physical processes, which could significantly influence oil dissolution rates. Furthermore, the absence of comparative data from other similar marine ecosystems challenges the determination of whether the observed rates are specific to the Red Sea region. To capture the full spectrum of temporal variability in oil dissolution rates and associated microbial responses, extended-duration incubation experiments involving various types of crude oil samples would be helpful. Furthermore, future studies should incorporate exposure to the lowest, average, and highest temperature levels prevalent in the study area. This approach would help stimulate realistic environmental conditions and demonstrate the impact of ambient shifts in SST on oil degradation processes. Understanding how the short-term processes observed in this study may translate into broader ecological consequences holds significance for evaluating the overall resilience of the Red Sea to oil pollution.

## Conclusions

Crude oil trapped in coastal sediments is susceptible to many degradation processes releasing weathered oil components into the marine environment. The interaction among microbial degradation, photooxidation, and other physical weathering processes significantly influences their impacts on coastal habitats. Studies conducted in the Arabian Gulf (Saeed et al. 2011) and the Gulf of Mexico (Bacosa et al. 2015) demonstrated the stimulatory effects of environmental factors on the photodegradation of crude oil in the open ocean. Here, we presented compelling evidence for the first time showing that an interactive effect of high temperature and intense sunlight in the Red Sea can promote the ocean-scale processes of crude oil dissolution and release of oil carbon into the marine DOM pool. Employing a combination of high-resolution techniques, we demonstrate a significant conjoint effect of sunlight and high temperature in the Red Sea on the weathering and turnover of crude oil carbon into the seawater and a subsequent transformation of the indigenous bacterial flora into an HNA-dominated community. However, bacterial response and oil degradation potential in the highly oligotrophic Red Sea are likely limited by nutrient availability; therefore, nutrient amendment can substantially influence the oil-degrading bacterial processes. It was evident that the oil-derived fluorophores were not biologically degraded at the same rate as they were released and may remain as a major component of the DOM pool.

The present study provides baseline information on factors contributing to dissolution of crude oil on the surface of the Red Sea which is important in understanding and modeling fate of spilled oil in the region. Further investigations are recommended to reveal the probable consequences of these components on bacterial community dynamics and the potential long-term ecological consequences of these changes in the marine environment. Moreover, the methods described in this study are unlaborious, sensitive, and time-effective; therefore, their use in post-incident oil spill impact assessments and field studies on the fate and complex transformation processes of crude oil in the oceanic environment is promising.

### Supplementary Information

Below is the link to the electronic supplementary material.

Supplementary file1 (DOCX 18747 KB)

## Data Availability

All data are provided in the manuscript.
