# Supplementary material for: High temperature and solar radiation in the Red Sea enhance the dissolution of crude oil from surface films
Source: Environ Sci Pollut Res Int. 2024 Jun 10;31(29):42034–48. doi: 10.1007/s11356-024-33864-z (PMC11219460; doi:10.1007/s11356-024-33864-z)

**Supplementary Materials**

High temperature and solar radiation in the Red Sea enhance the dissolution of crude oil from surface films

Sreejith Kottuparambil^1, 2^*****, Ananya Ashok^1^, Patricia López^3^, Maan H. Amad^3^, Carlos M. Duarte^1, 4^ and Susana Agusti^1^

^1^Red Sea Research Center (RSRC), King Abdullah University of Science and Technology (KAUST), Thuwal 23955-6900, Saudi Arabia.

^2^ Mubadala Arabian Center for Climate and Environmental Sciences (ACCESS), New York University Abu Dhabi, Abu Dhabi, United Arab Emirates.

^3^ King Abdullah University of Science and Technology, Core Labs, Thuwal 23955-6900, Saudi Arabia.

^4^Computational Bioscience Research Center (CBRC), King Abdullah University of Science and Technology, Thuwal 23955-6900, Saudi Arabia.

*Corresponding author:

E-mail: [sk11452@nyu.edu](mailto:sk11452@nyu.edu)

**List of Methods**

**Method S1.** Analysis of total petroleum hydrocarbons in crude oil

**Method S2.** Analysis of PAHs in crude oil sample

**Method S3.** Analysis of PAHs in seawater

**Method S4.** Flow cytometric enumeration of HNA and LNA bacteria

**List of Tables**

**Table S1.** Retention time (RT), internal standards (ISTD), transitions and collision energies (CE) used for the determination of PAHs

**Table S2.** Total Petroleum Hydrocarbons, total carbon and PAHs concentrations in crude oil and seawater

**Table S3.** Two-way analysis of variance (ANOVA) for various variables

**List of Figures**

**Figure S1.** Map showing oil slick after the 2019 Sabiti oil spill in the Red Sea and the oil sample collection site at Al Saif beach, near the port city of Jeddah, Saudi Arabia.

**Figure S2.** Experimental apparatus

**Figure S3.** Levels of natural solar radiation and UV radiation during incubation

**Figure S4.** Increase in the area under integrated light oil fluorescence curve

**Figure S5.** Initial and final proportions of high nucleic acid (HNA) and low nucleic acid (LNA) bacteria in seawater

**Figure S6.** Spearman’s correlation heatmap showing relationship among various parameters

**Supplementary Methods**

***Method S1.*** *Analysis of total petroleum hydrocarbons (TPH; C_8_−C_40_) in crude oil*

Briefly, a 10 g sample was ground with anhydrous sodium sulfate and extracted by accelerated solvent extraction (ASE) using dichloromethane at 15 psi. The resulting extract was evaporated using DryVap automatic concentrator (Horizon, USA) and reconstituted in dichloromethane (10 mL). This extract was cleaned with dispersive solid-phase extraction (dSPE) and a 1 mL aliquot was transferred to GC vial and analyzed by GC-FID (Agilent 7890, Agilent Technologies, Santa Clara, CA, USA). The calibration curve concentration ranged from 85 – 5100 mg kg^−1^ and the limit of quantification was 3 mg kg^−1^. Quality Assurance and Quality Control (QA/QC) included solvent, digestion blanks, and clean sediment spiked with TPH standards at 1700 ppm with a recovery rate of 68%.

***Method S2.*** *Analysis of PAHs in crude oil sample*

Levels of 18 different PAHs (16 EPA PAH, 1-methylnapthalene, and 2-methylnapthalene) in the crude oil sample collected at the Red Sea coast were measured by accelerated solvent extraction (ASE) using dichloromethane as solvent followed by gas chromatography tandem Triple-Quadrupole mass spectrometry (GC-MS/MS, Agilent 7890 GC 7010B/MS, Agilent Technologies, Santa Clara, CA, USA). Briefly, 5 g of oiled sediment (with approximately 1 g of oil) was dried using sodium sulfate and extracted in dichloromethane by ASE. A small amount of activated copper powder was added to the extract to remove any elemental sulfur and the extract was cleaned up through a silica-gel solid-phase extraction (SPE) using the EnvirElut-PAH column. After concentrating using a Genevac rocket evaporator (SP, Warminster, PA), the extract was reconstituted with 2 mL dichloromethane. PAHs were analyzed by GC-MS/MS using a DB-EU PAH 20 m. 0.18 mm, 0.14 μm film thickness column, and HP-5MS (30 m, 0.25 mm, 0.25 μm). Quantification was performed by an external calibration curve of PAHs ranging from 1 ng/mL to 250 ng/mL in

dichloromethane with an internal standard of 100 ng/mL. This method included specific quality control parameters that are applicable to the US-EPA Method 8270 D (USEPA, 1998).

***Method S3.*** *Analysis of PAHs in seawater*

PAHs in seawater were analyzed by solid-phase extraction (SPE) using an EnvirElut-PAH cartridge (1 gm/6 mL, Agilent, Santa Clara, CA), followed by GC-MS/MS (Agilent 7890 GC 7010B/MS, Agilent Technologies, Santa Clara, CA, USA). Briefly, the thawed water samples were mixed vigorously and 25 mL of aliquots were cleaned up by SPE using the EnvirElut-PAH column. The SPE columns were previously conditioned with methanol and Milli-Q water. PAHs were eluted with 12 mL cyclohexane. One mL of toluene was added as keeper to the eluates, which were further concentrated down to 1 mL using a Genevac rocket evaporator (SP, Warminster, PA). One µL of the extract was injected into a DB-EU PAH 20 m. 0.18 mm, 0.14 μm film thickness column. Quantification of total PAHs was done by an external calibration curve of PAHs ranging from 1 ng mL^−1^ to 100 ng mL^−1^ in dichloromethane with internal PAH standard at 100 ng mL^−1^. PAHs were confirmed by comparing retention times and ion ratios between sample extracts and standards, with a practical quantitation limit (PQL) of 0.04 μg L^−1^. The average percentage recovery of individual PAHs was 83%.

***Method S4.*** *Flow cytometric enumeration of HNA and LNA bacteria*

Abundances of HNA and LNA bacterial groups were estimated using the BD FACSCanto II flow cytometer (BD Biosciences, Eysins, Switzerland). Briefly, samples were thawed and 400 µL was transferred to a SIP (sample-injection-port) tube. Bacterial cells were stained with 1% v/v SYBR Green and incubated in dark at room temperature (~23 °C) for 15 minutes. Prior to counting, 10 µL of homogenous suspension of fluorescent microbeads (1µm diameter, 1 x 10^6^ beads/mL) was added to the stained sample. The beads solution was sonicated for 15 mins prior to addition to mix the suspension homogenously.

Noise was identified without any thresholds before setting a sample-acquisition threshold of 200 on FITC-A. Samples were acquired at low flow speed for 60 seconds or until 10,000 events were recorded. A SIT flush was performed automatically between samples. Daily after fluidics start-up, the flow cell was cleaned with FACS-Clean Solution and by running MilliQ water for 5 mins prior to samples acquisition. The flowrate was gravimetrically calculated daily by running a pre-weighed SIP tube containing 1 mL of MilliQ water at low flow-speed for 5 minutes. The tube was weighed after, and the difference in weights divided by acquisition time was used to calculate the daily flowrate.

HNA-LNA populations were distinguished based on their side scatter and green fluorescence signal (SSC-H vs. FITC-A 4-log decade dot plots). Bacterial cell abundance of individual nucleic acid groups was calculated based on the flow rate, compensating for the dilution effect of fixative, stain, and beads solutions. The data were batch-processed using the FCS Express 6 RUO software (DeNovo Software, Glendale, CA). A SIT flush was performed automatically between samples. Daily after fluidics start-up, the flow cell was cleaned with FACS-Clean Solution and by running MilliQ water for 5 mins prior to samples acquisition.

Bacterial growth rates were obtained by the following formula:

Growth rate (k) = (LN_T1_ – LN_T0_)/1

Where T1 is the initial bacterial abundance and T0 is the final bacterial abundance.

**Supplementary Tables**

**Table S1.** Retention time (RT), internal standards (ISTD), transitions and collision energies (CE) used for the determination of PAHs.

| **Compound Name** | **RT^a^** | **Associated ISTD^b^** | **Transitions** | **CE (eV)** |
| --- | --- | --- | --- | --- |
| Naphthalene | 3.06 | Naphthalene-d_8_ | 128.0 > 102.0  128.0 > 127.0 | 22  20 |
| 2-Methylnaphthalene | 3.35 | Naphthalene-d_8_ | 142.0 > 115.0  142.0 > 141.0 | 30  30 |
| 1-Methylnaphthalene | 3.44 | Naphthalene-d_8_ | 142.0 > 115.0  142.0 > 141.0 | 30  30 |
| 2-Fluorobiphenyl | 3.55 | Acenaphthene-d_10_ | 172.0 > 169.9  172.0 >150.9 | 25  25 |
| Acenapthylene | 4.05 | Acenaphthene-d_10_ | 152.0 > 150.0  152.0 >150.9 | 40  40 |
| Acenapthene | 4.15 | Acenaphthene-d_10_ | 154.0 > 152.0  153.0 > 152.0 | 40  40 |
| Fluorene | 4.60 | Acenaphthene-d_10_ | 166.0 > 165.0  166.0 >163.0 | 30  33 |
| Phenanthrene | 6.23 | Phenanthrene-d_10_ | 178.1 > 176.0  178.1 > 152.1 | 34  25 |
| Anthracene | 6.32 | Phenanthrene-d_10_ | 178.1 > 176.0  178.1 > 152.1 | 34  25 |
| Fluoranthene | 9.98 | Phenanthrene-d_10_ | 202.0 > 201.0  202.0 > 200.0 | 50  50 |
| Pyrene | 11.22 | Chrysene-d_12_ | 202.0 > 201.0  202.0 >200.0 | 30  30 |
| 4-Terphenyl-d_14_ | 11.54 | 2,4-DDT-d_8_/ Chrysene-d_12_ | 244.0 > 242.2  244.0 > 240.2  244.0 >212.0 | 18  30  10 |
| Benz(a)anthracene | 18.01 | Chrysene-d_12_ | 228.0 > 226.0  228.0 > 226.0 | 38  38 |
| Chrysene | 18.52 | Chrysene-d_12_ | 228.0 > 226.0  228.0 > 226.0 | 38  38 |
| Benzo(b)fluoranthene | 25.33 | Perylene-d_12_ | 252.0 > 250.0  250.0 > 248.0 | 42  40 |
| Benzo(k)fluoranthene | 25.52 | Perylene-d_12_ | 252.0 > 250.0  250.0 > 248.0 | 42  40 |
| Benzo(a)pyrene | 27.97 | Perylene-d_12_ | 252.0 > 250.0  250.0 > 248.0 | 40  40 |
| Indeno(1,2,3,-cd)pyrene | 34.43 | Perylene-d_12_ | 276.0 > 274.0  276.0 > 247.0  276.0 > 276.0 | 42  60  15 |
| Dibenz(a,h)anthracene | 34.62 | Perylene-d_12_ | 278.0 > 276.0  278.0 > 252.0  278.0 > 278.0 | 38  60  15 |
| Benzo(g,h,i)perylene | 35.99 | Perylene-d_12_ | 274.0 > 272.0  277.0 > 275.0  276.0 > 274.0 | 42  38  42 |
| Naphthalene-d_8_ | 3.04 |  | 136.1 > 108.1  136.1 > 84.1 | 20  25 |
| Acenaphthene-d_10_ | 4.11 |  | 162.0 > 160.0  164.1 > 162.1 | 19  15 |
| Phenanthrene-d_10_ | 6.19 |  | 188.3 > 186.3  188.3 > 160.2 | 15  20 |
| Chrysene-d_12_ | 18.40 |  | 240.0 > 236.0  118.0 > 116.0 | 25  25 |
| Perylene-d_12_ | 28.61 |  | 264.0 > 260.0  264.0 > 236.0 | 40  25 |

^a^RT are indicative. They are subject to change depending on the column trimming

**Table S2.** Total Petroleum Hydrocarbons (TPH), total carbon and PAHs concentrations in the crude oil used in this study and in seawater before and after 4-d incubations. NA= not analyzed.

| PAH | Crude oil  (μg kg^−1^) | Seawater (μg L^−1^) | | | | | | | | |
| --- | --- | --- | --- | --- | --- | --- | --- | --- | --- | --- |
|  |  | Initial | **Treatment/** **incubation temperature (± 0.5 °C)** | | | | | | | |
|  |  |  | SWC 26 | SWC 30 | D 26 | D 30 | RSR 26 | RSR 30 | FSR 26 | FSR 30 |
| 1-methylnaphthalene | 345 | <PQL | <PQL | <PQL | <PQL | <PQL | <PQL | <PQL | **0.16** | <PQL |
| 2-methylnaphthalene | 247 | <PQL | <PQL | <PQL | <PQL | <PQL | <PQL | <PQL | **0.20** | <PQL |
| Acenapthene | 1438 | <PQL | <PQL | <PQL | <PQL | <PQL | <PQL | <PQL | <PQL | <PQL |
| Acenapthylene | 2959 | <PQL | <PQL | <PQL | <PQL | <PQL | <PQL | <PQL | <PQL | <PQL |
| Anthracene | 2502 | <PQL | <PQL | <PQL | <PQL | <PQL | <PQL | <PQL | <PQL | <PQL |
| Benzo(a)anthracene | 910 | <PQL | <PQL | <PQL | <PQL | <PQL | <PQL | <PQL | <PQL | <PQL |
| Benzo(a)pyrene | 462 | <PQL | <PQL | <PQL | <PQL | <PQL | <PQL | <PQL | <PQL | <PQL |
| Benzo(b)fluoranthrene | 1099 | <PQL | <PQL | <PQL | <PQL | <PQL | <PQL | <PQL | <PQL | <PQL |
| Benzo(g,h,i)perylene | 471 | <PQL | <PQL | <PQL | <PQL | <PQL | <PQL | <PQL | <PQL | <PQL |
| Benzo(k)fluoranthrene | 251 | <PQL | <PQL | <PQL | <PQL | <PQL | <PQL | <PQL | <PQL | <PQL |
| Chrysene | 15824 | <PQL | <PQL | <PQL | <PQL | <PQL | <PQL | <PQL | <PQL | <PQL |
| Dibenz(a,h)anthracene | 219 | <PQL | <PQL | <PQL | <PQL | <PQL | <PQL | <PQL | <PQL | <PQL |
| Fluoranthene | 3626 | **0.08** | **0.08** | **0.09** | **0.08** | **0.08** | **0.09** | **0.09** | **0.09** | **0.1** |
| Fluorene | 6598 | <PQL | <PQL | <PQL | **0.12** | **0.11** | **0.14** | **0.17** | **0.33** | **0.16** |
| Indeno(1,2,3,-cd)pyrene | 106 | <PQL | <PQL | <PQL | <PQL | <PQL | <PQL | <PQL | <PQL | <PQL |
| Napthalene | <PQL | <PQL | <PQL | <PQL | <PQL | <PQL | <PQL | <PQL | **0.30** | <PQL |
| Phenanthrene | 26073 | **0.13** | **0.13** | **0.14** | **0.28** | **0.20** | **0.47** | **0.62** | **0.52** | **0.58** |
| Pyrene | 2082 | <PQL | <PQL | <PQL | <PQL | <PQL | <PQL | <PQL | <PQL | <PQL |
| TPH (mg kg^−1^) | 146,580 | NA | NA | NA | NA | NA | NA | NA | NA | NA |
| Total carbon (%) | 47.1 | NA | NA | NA | NA | NA | NA | NA | NA | NA |
| Total hydrogen (%) | 9.1 | NA | NA | NA | NA | NA | NA | NA | NA | NA |

**Table S3.** Two-way analysis of variance (ANOVA) for various variables in seawater incubated for 4 days with crude oil film at two temperatures and solar radiation levels (DF: Degrees of freedom, F ratio: the model mean square divided by the error mean square, Prob > F: the *p*-value for the test).

| Source | DF | F ratio | Prob > F |
| --- | --- | --- | --- |
| Total Organic Carbon (TOC) |  |  |  |
| Temperature (A) | 1 | 12.37 | 0.0042 |
| Solar radiation (B) | 2 | 222.38 | <0.0001* |
| Interaction of A × B | 2 | 3.40 | 0.0675 |
| Oil degradation rate |  |  |  |
| Temperature (A) | 1 | 6.63 | 0.0244 |
| Solar radiation (B) | 2 | 2E+06 | <0.0001 |
| Interaction of A × B | 2 | 1E+05 | <0.0001 |
| CDOM absorption (a_254_) |  |  |  |
| Temperature (A) | 1 | 1.91 | 0.192 |
| Solar radiation (B) | 2 | 12.03 | 0.0013 |
| Interaction of A × B | 2 | 0.04 | 0.9641 |
| Light oil fluorescence |  |  |  |
| Temperature (A) | 1 | 21.78 | <0.0001 |
| Solar radiation (B) | 2 | 4.74 | 0.0304 |
| Interaction of A × B | 2 | 1.05 | 0.3804 |
| Heavy oil fluorescence |  |  |  |
| Temperature (A) | 1 | 32.24 | 0.0001 |
| Solar radiation (B) | 2 | 50.81 | <0.0001 |
| Interaction of A × B | 2 | 7.87 | 0.0065 |
| HNA growth rate |  |  |  |
| Temperature (A) | 1 | 37.18 | <0.0001 |
| Solar radiation (B) | 2 | 86.58 | <0.0001 |
| Interaction of A × B | 2 | 27.83 | <0.0001 |
| δ^13^C-CH_4_ |  |  |  |
| Temperature (A) | 1 | 0.12 | 0.7375 |
| Solar radiation (B) | 2 | 33.76 | <0.0001 |
| Interaction of A × B | 2 | 0.11 | 0.8991 |
| δ^13^C-CO_2_ |  |  |  |
| Temperature (A) | 1 | 26.76 | 0.0002 |
| Solar radiation (B) | 2 | 322.34 | <0.0001 |
| Interaction of A × B | 2 | 29.87 | <0.0001 |

**Supplementary Figures**

**Figure S1:** Map showing oil slick after the 2019 Sabiti oil spill in the Red Sea and the oil sample collection site at Al Saif beach, near the port city of Jeddah, Saudi Arabia. Red triangle indicates the position of the Sabiti oil tanker during the spill event on 11 October 2019.

**
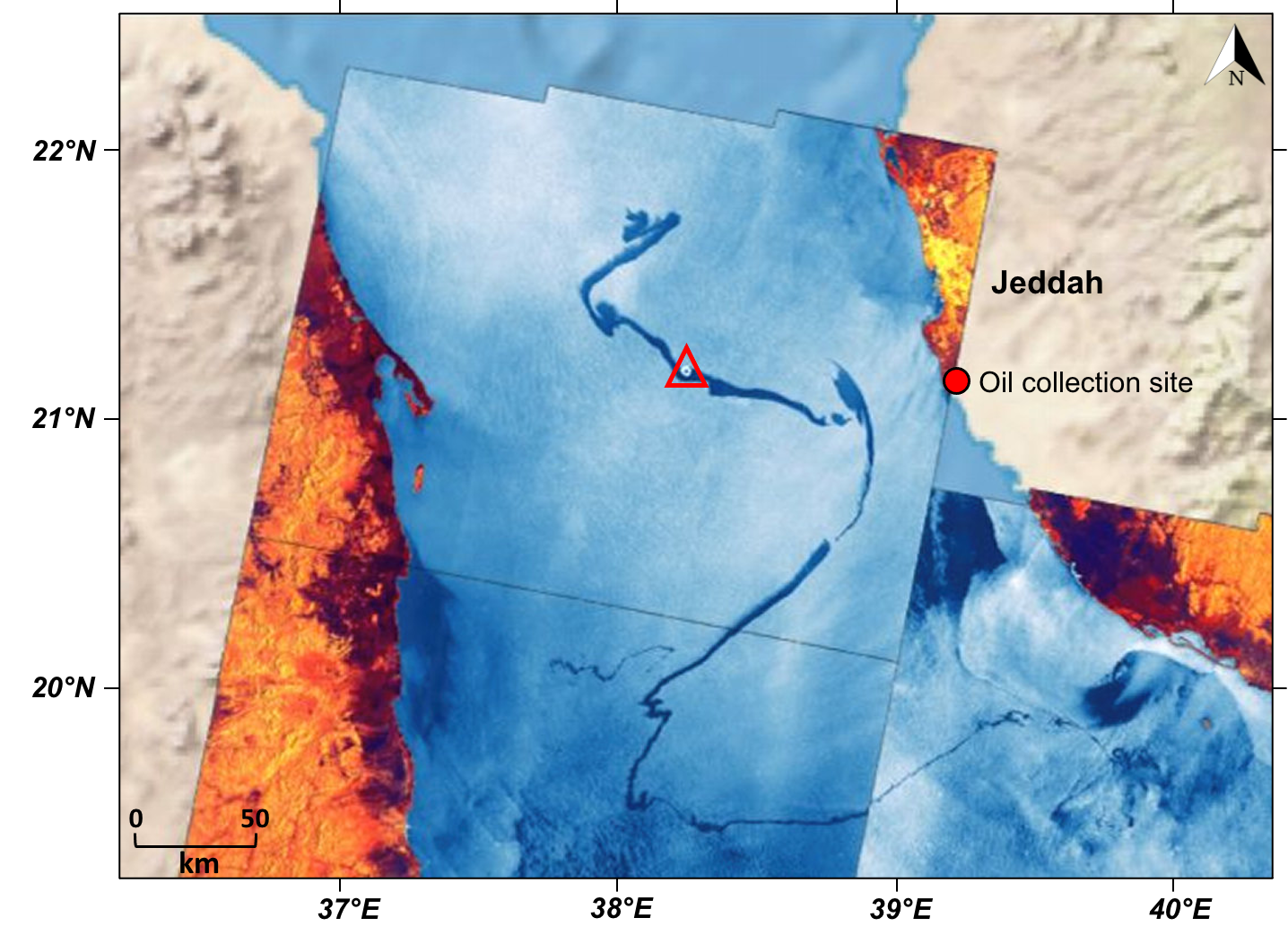
**

**Figure S2.** Experimental apparatus.

**
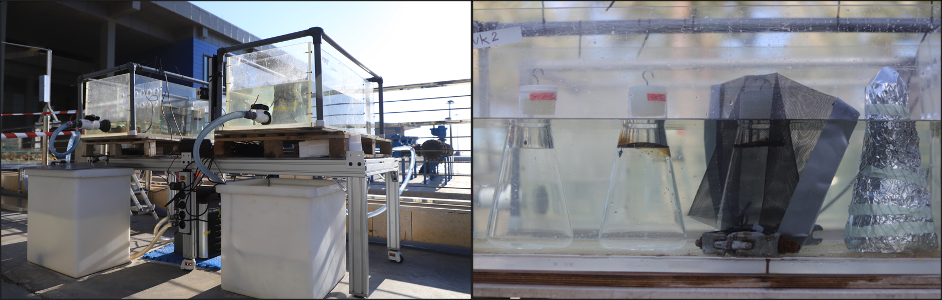
**

**Figure S3.** Levels of incident natural solar radiation and UV radiation during the 4-d incubation of Red Sea water with and without crude oil surface films.


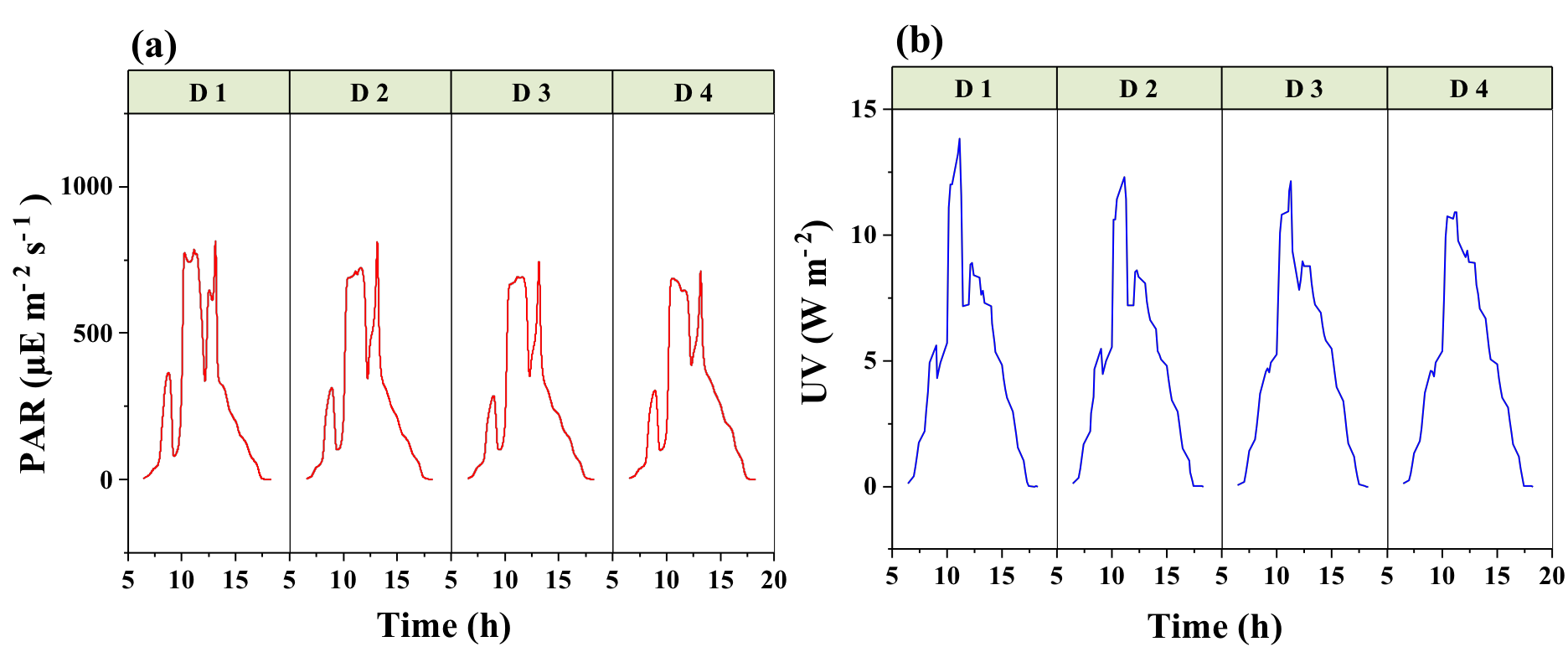


**Figure S4.** Increase in the area (fold increase from the seawater control) under integrated light oil fluorescence curve on day 4 in seawater during incubation with or without crude oil films. D = treatment with an oil film in dark, RSR = treatment with an oil film under 50% incident solar radiation, FSR = treatment with an oil film under full incident solar radiation.


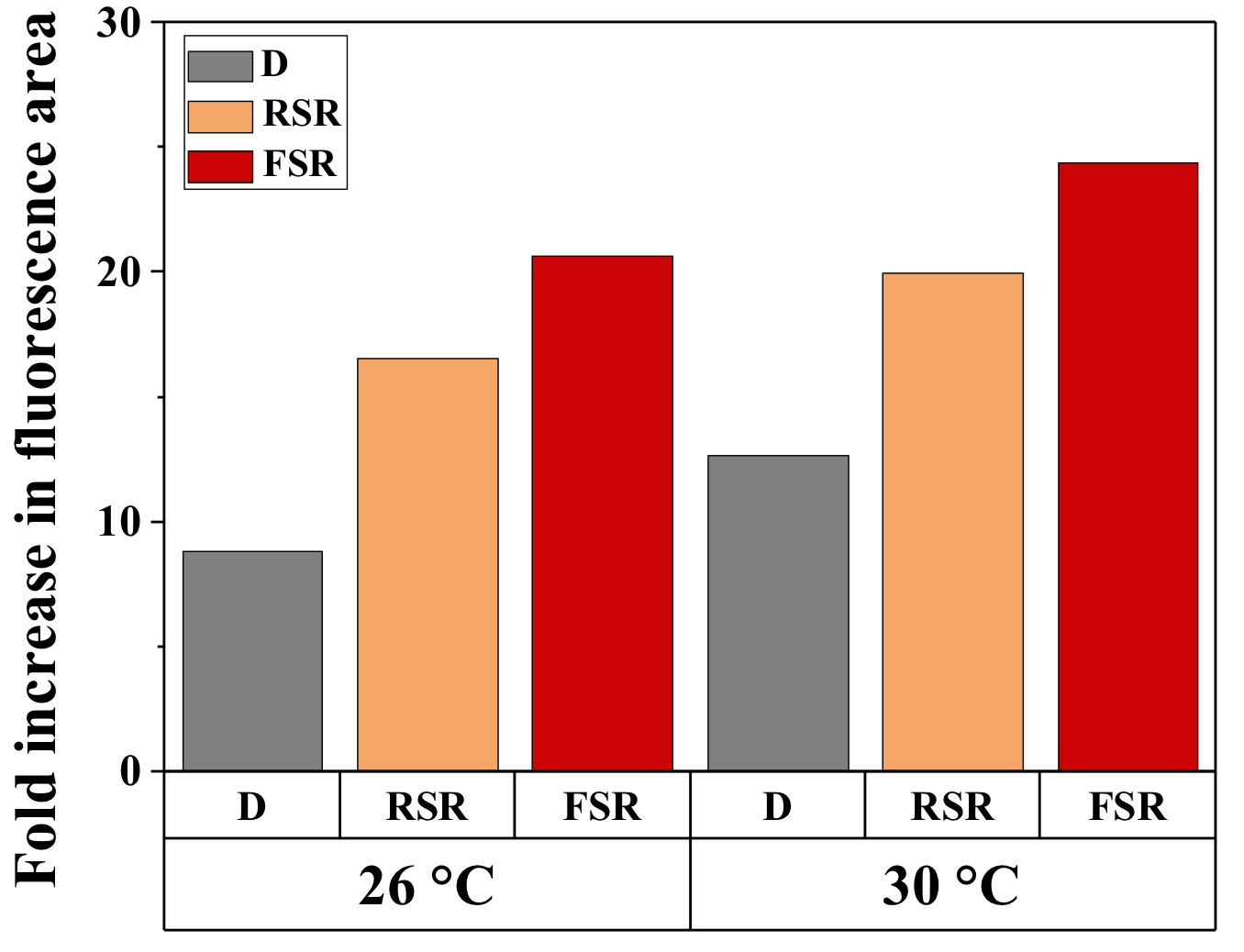


**Figure S5.** Initial and final proportions of high nucleic acid (HNA) and low nucleic acid (LNA) bacteria in seawater after 4-d incubation with or without crude oil film at 26 °C (a) and 30 °C (b). SWC = seawater control without an oil film under full solar radiation, D = treatment with an oil film in dark, RSR = treatment with an oil film under 50% solar radiation, FSR = treatment with an oil film under full solar radiation.

**Figure S6.** Spearman’s correlation heatmap showing relationship among changes in Total Organic Carbon (TOC) levels, CDOM absorption (a_254_), and HNA bacteria growth rate measured in seawater after 4-d incubation in the presence of crude oil surface films.


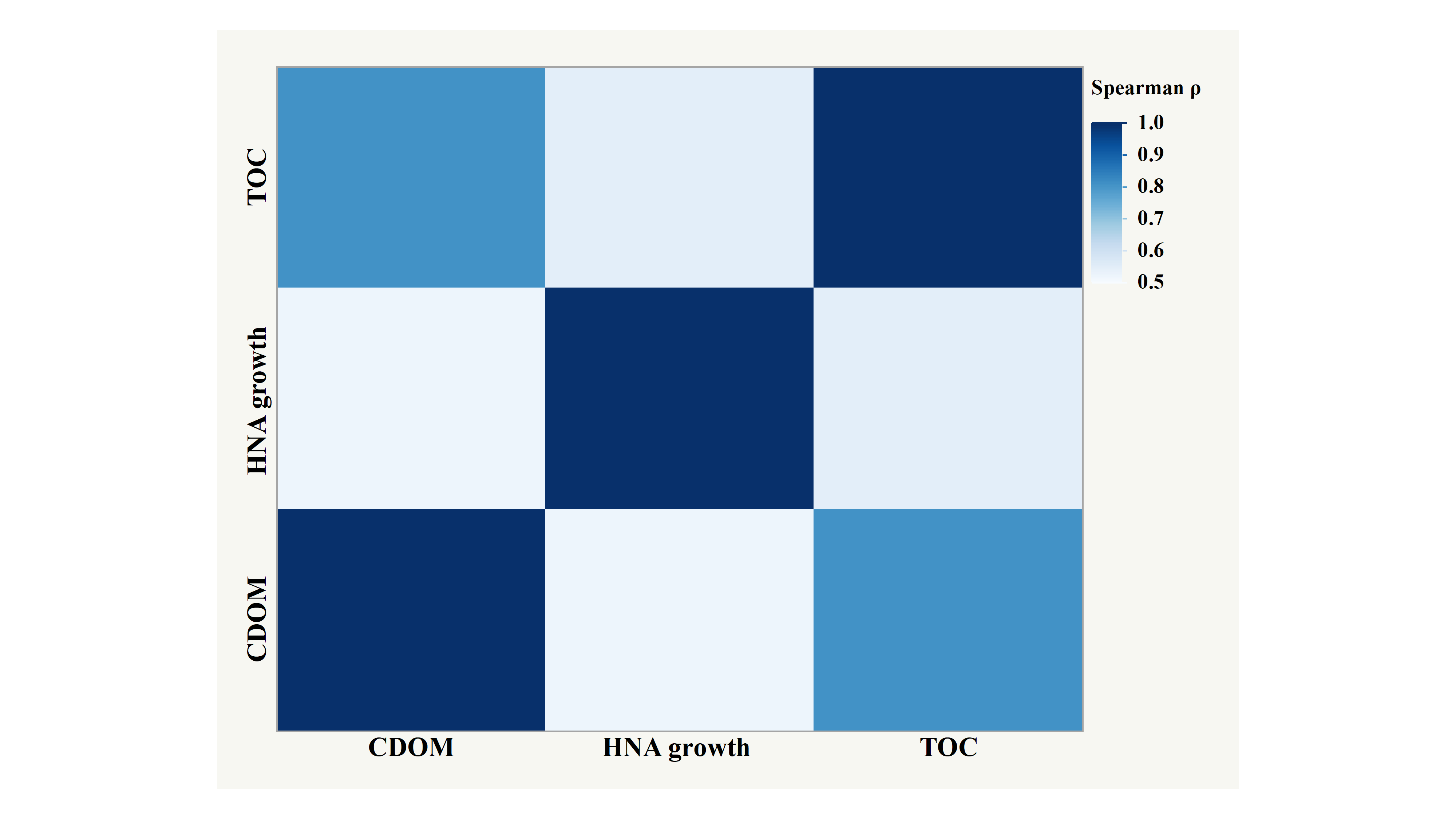

Supplement: Supplementary file 1 — Supplementary file1 (DOCX 18747 KB) [file 11356_2024_33864_MOESM1_ESM.docx]
